# Oral Microbiome Diversity Matters on Nucleos(t)ide Analogue Cessation in Chronic Hepatitis B

**DOI:** 10.1093/infdis/jiaf591

**Published:** 2025-12-02

**Authors:** Mahin Ghorbani, Agne Kvedaraite, Khaled Al-Manei, Choon Boon Too, Susanne Cederberg, Asgeir Johannessen, Dag Henrik Reikvam, Davide Valentini, Christopher Maucourant, Niklas K Björkström, Soo Aleman, Margaret Sällberg Chen

**Affiliations:** Division of Pathology, Department of Laboratory Medicine, Karolinska Institutet, Huddinge, Sweden; Division of Pathology, Department of Laboratory Medicine, Karolinska Institutet, Huddinge, Sweden; Folktandvården Stockholm, Sweden; Division of Pathology, Department of Laboratory Medicine, Karolinska Institutet, Huddinge, Sweden; Division of Endodontics, Department of Restorative Dental Science, College of Dentistry, King Saud University, Riyadh, Saudi Arabia; Division of Pathology, Department of Laboratory Medicine, Karolinska Institutet, Huddinge, Sweden; Department of Infectious Diseases, Karolinska University Hospital, Stockholm, Sweden; Department of Infectious Diseases, Vestfold Hospital Trust, Tønsberg, Norway; Department of Infectious Diseases, Regional Advisory Unit for Imported and Tropical Diseases, Oslo University Hospital, Oslo, Norway; Faculty of Medicine, Institute of Clinical Medicine, University of Oslo, Oslo, Norway; Department of Infectious Diseases, Regional Advisory Unit for Imported and Tropical Diseases, Oslo University Hospital, Oslo, Norway; Faculty of Medicine, Institute of Clinical Medicine, University of Oslo, Oslo, Norway; Department of Cellular Therapy and Allogeneic Stem Cell Transplantation, Karolinska University Hospital Huddinge and Karolinska Comprehensive Cancer Center, Stockholm, Sweden; Center for Infectious Medicine, Department of Medicine Huddinge, Karolinska Institutet, Karolinska University Hospital, Huddinge, Sweden; Center for Infectious Medicine, Department of Medicine Huddinge, Karolinska Institutet, Karolinska University Hospital, Huddinge, Sweden; Department of Infectious Diseases, Karolinska University Hospital, Stockholm, Sweden; Center for Infectious Medicine, Department of Medicine Huddinge, Karolinska Institutet, Karolinska University Hospital, Huddinge, Sweden; Division of Pathology, Department of Laboratory Medicine, Karolinska Institutet, Huddinge, Sweden; Department of Cellular Therapy and Allogeneic Stem Cell Transplantation, Karolinska University Hospital Huddinge and Karolinska Comprehensive Cancer Center, Stockholm, Sweden

**Keywords:** viral hepatitis, oral microbiome, antiviral treatment, nucleos(t)ide analogue discontinuation, HBV, cure

## Abstract

**Background:**

Withdrawal of nucleos(t)ide analogue (NUC) therapy in hepatitis B e antigen (HbeAg)-negative chronic hepatitis B (CHB) may lead to functional cure in a subset of patients. Although gut microbiota is known to influence both CHB progression and treatment outcomes, the oral microbiome in NUC cessation remains unexplored.

**Methods:**

This longitudinal study explored the oral microbiome in patients with CHB on NUC therapy > 2 years having a planned NUC cessation. Oral microbiome composition was analyzed in 110 saliva samples across 7 time points from 18 HBeAg-negative patients with 36 months follow-up. Favorable outcome was defined as either HBsAg loss or decline of > 1 log_10_ or sustained off-therapy HBV DNA level < 2000 IU/mL during year 3. Hepatic flare was defined as alanine transaminase (ALT) > 80 U/L or 2 × baseline level.

**Results:**

The overall microbial composition remained stable during the study period. Patients with favorable outcome showed consistently higher alpha diversities (*P* < .001) from baseline, with lower intersample variations across all time points (*P* < .05), compared to unfavorable. Hepatitis B surface antigen (HBsAg), ALT, and aspartate transaminase (AST) correlated inversely with several *Prevotella* taxa and specific pathways (Spearman ρ > −0.5, *P* < .01). Unfavorable outcome and high HBsAg level correlated with opportunistic taxa *Haemophilus parainfluenzae* and *Porphyromonas catoniae*. Random forest model incorporating validated microbial markers predicting favorable versus unfavorable outcome achieved higher predictive performance than clinical markers alone (area under curve, 0.79 vs 0.66).

**Conclusions:**

Our exploratory study suggests that oral microbiome profiling at NUC cessation in HBeAg-negative CHB could support prognostication of virological outcome.

**Clinical Trials Registration**. NCT03681132.

## Teaser

This 3-year longitudinal study identifies a novel association between the oral microbiome profile and the clinical outcome after withdrawal of nucleos(t)ide analogues in chronic hepatitis B, indicative of potential applications in oral microbiome-based prediction and development of personalized treatment strategies.

Chronic hepatitis B (CHB) remains a significant global health challenge, affecting about 296 million people. With a global hepatitis B surface antigen (HBsAg) seroprevalence rate of 3.8%, these individuals face substantial risk of developing cirrhosis and hepatocellular carcinoma [1]. Nucleos(t)ide analogues (NUCs) are first-line therapies that suppress viral replication, reduce liver injury, and halt disease progression [2–4]. While NUCs effectively suppress viral load, they rarely lead to HBsAg loss, the hallmark of a functional cure [5].

Recently, interest has grown in NUC discontinuation, particularly in hepatitis B e antigen (HbeAg)-negative CHB patients with mild liver disease [6, 7]. Stopping NUC therapy may promote functional cure by triggering immune reactivation but also carries risk of hepatic flares [8, 9]. Reported risk factors for relapse include older age, male sex, higher end-of-treatment HBsAg levels, and tenofovir-based therapy [10]. Emerging data indicate that the likelihood of favorable off-therapy outcomes increases over time, although mechanisms underlying spontaneous HBsAg clearance remain unclear.

The gut-liver axis and microbiome interactions can influence CHB progression, antiviral treatment response, and immune modulation [11–14]. Reduced microbial richness and diversity manifest in changes of specific oral taxa and reflect functional and ecological microbiome disturbances. The oral microbiome´s involvement in gut and intestine microbiome cross-niche microbial overlap has been frequently observed in advanced cirrhosis [15–18]. In contrast, patients with CHB with stable cirrhosis or no liver injury often show oral microbiomes resembling healthy- or vaccine-protected individuals [18, 19], suggesting oral microbiome may serve as a proxy marker of liver health.

Oral dysbiosis contributes to systemic endotoxemia and immune activation in liver cirrhosis and hepatic encephalopathy [18, 20, 21]. For example, in patients with cirrhosis, integrating salivary microbiome data into biomarker panels improves prediction of hospitalizations [22]. Modifying the periodontal microbiome has reduced systemic inflammation and improve model for end-stage liver disease (MELD) scores and cognitive function [23]. Mechanistic evidence supports that dysbiotic translocation of oral pathobionts, pathogen-associated molecular patterns (PAMPs), and proinflammatory mediators can potentiate systemic immune activation and amplify ongoing inflammation through the oral-gut-liver axis [21, 24]. This emerging insight offers new conceptual perspectives for preventing CHB progression and supporting a functional HBV cure.

Although oral microbiome changes are implicated in CHB, no studies have investigated its role in the context of NUC withdrawal or clinical outcomes. Our study aimed to explore oral microbiome diversity in patients with CHB undergoing NUC discontinuation. This 3-year longitudinal follow-up represents the first oral microbiome dataset in this underexplored area of hepatitis B research.

## METHODS

### Patient Recruitment

The study was conducted as part of an open-label, randomized, and multicenter clinical trial (EudraCT No., 2018-000724-34; https://clinicaltrials.gov/study/NCT03681132?cond=NCT03681132&rank=1, NCT03681132) between October 2019 and February 2023. In this substudy, 18 adult patients (aged 18–70 years) with HbeAg-negative CHB, at least 2 years of NUC therapy, and full viral suppression were included. Patients with history of cirrhosis or hepatocellular carcinoma were excluded, and all included had a Child-Pugh class A5 score. At cessation of NUC therapy during October 2019 to February 2020, the patients were randomized into 2 restart strategies (high or low threshold for restart), as previously published [25]. The participants were recruited from the Department of Infectious Diseases, Karolinska Hospital, Sweden. Baseline was set to the date of NUC cessation. A total of 7 time points over 36 months of follow-up were evaluated.

The patients were classified depending on their virological and biochemical evolution after NUC cessation. Favorable outcome was defined as either HBsAg loss or decline of > 1 log_10_ IU/mL from baseline to 36 months, or HBV DNA level of < 2000 IU/mL at all study visits during 24–36 months, without any retreatment. Hepatic flare was defined as presence of an increase in alanine transaminase (ALT) level to > 80 U/L (equal to > 1.33 microkat/L), or 2 × baseline level after NUC cessation.

Written informed consent was obtained for all patients. This study was approved by the Swedish Authority of Ethics (reference number Dnr: 2019-02725) and conducted according to the Declaration of Helsinki and International Conference on Harmonization Good Clinical Practice.

### Biochemical and Virological Analyses

Analyses of biochemical tests were performed at Karolinska University Hospital Laboratory. The HBV DNA test had a detection limit of 10 IU/mL. Quantitative HBsAg analysis was performed in 1 batch with the Elecsys HBsAg II Quant assay (Roche Diagnostics) on the Roche Cobas e801 platform, a 2-step sandwich chemiluminescent microparticle immunoassay (detection limit 0.05 IU/mL).

### Oral Microbiome Sampling and Sequencing

Unstimulated morning saliva samples were collected as previously described with a questionnaire for oral health and lifestyle [19, 26]. Samples were transferred immediately to laboratory, vortexed, and aliquoted into sterile tubes for storage at −80°C within 2 hours after collection. Salivary DNA extractions was performed using EZ1 Advanced XL automated workstation (Qiagen) with EZ1 Advanced XL DNA Bacteria Card (catalog No. 9018694; Qiagen) and EZ1&2 DNA Tissue Kit, eluted in 100 μL RNase-free water, and stored at −20°C until further processing. Extracted DNA was assessed by NanoDrop spectrophotometer and Qubit fluorometer (Thermo Fisher Scientific) and confirmed by 1% agarose gel electrophoresis. Only samples with 260/280 nm absorbance ratios between 1.8 and 2.0 and 260/230 nm absorbance ratios exceeding 2.0 were retained for library preparation.

Amplicon library preparation and sequencing were performed by Novogene UK following standard protocols, Illumina NovaSeq 6000 platform, and the 2 × 250 bp paired-end format targeting the V3–V4 region of the 16S rRNA gene (approximately 460 bp) using barcoded primers (16S, CCTAYGGGRBGCASCAG and GGACTACNNGGGTATCTAAT). Equimolar pooling of purified polymerase chain reaction (PCR) products was used to reduce intersample bias during sequencing. The pooled products underwent end repair, A tailing, and ligation with Illumina-compatible indexed adapters. Final libraries were purified, assessed for size distribution using a microfluidic-based bioanalyzer, and quantified using fluorometric methods and real-time PCR. Sequencing was conducted on an Illumina paired-end platform (PE250).

### Bioinformatics and Statistical Analysis

Sequencing generated a total of 7 271 154 raw paired end reads. Raw data were demultiplexed in QIIME2 (version 2022.2) [27], quality checked with FastQC (version 0.11.8) [28], and processed with Cutadapt (version 2.8) [29] to remove primers and residual adapter sequences. The DADA2 pipeline was applied for quality filtering, error correction, paired end read merging, and chimera removal. Posttrimming read lengths and 3′-end quality scores were examined to confirm sufficient overlap for accurate merging. Mean merged sequence length was approximately 424 bp with average overlap of 23 bp in DADA2 [30]. After DADA2 processing, 6 236 919 reads were retained corresponding to 5 503 300 bacterial amplicon sequence variants (ASVs). Taxonomic assignment of ASVs was performed in QIIME2 using the feature classifier classify-sklearn method with a pretrained naive Bayes classifier trained on SILVA database (release 138.1, 99% similarity clustered reference sequences) [31]. Confidence threshold of 0.7 was applied for classification from kingdom to species level.

Across the 110 saliva samples, 77% of ASVs were classified to species level and 23% remained unclassified. Classified ASVs had higher mean sequence frequencies (188 398 reads) than unclassified ASVs (217.5 reads), and their average classifier confidence was 0.95. This indicates that abundant and clinically relevant taxa were robustly classified, while unclassified features were rare and low frequency. ASV tables were subsequently prevalence filtered (minimum 2 counts, > 10% prevalence). Total sum scaling normalization was applied to account for variations in sequencing depth. Alpha diversity metrics (observed ASVs, Shannon diversity, Simpson index) were calculated using vegan (version 2.5) [32] in R (version 4.0.2). Beta diversity was estimated using Bray-Curtis dissimilarity and visualized via nonmetric multidimensional scaling using metaMDS function. Permutational multivariate analysis of variance (PERMANOVA) was conducted with adonis2 function (vegan) using 999 permutations. Statistical significance was defined as *P* < .05.

Differential microbial features associated with specific conditions were identified using linear discriminant analysis effect size (LEfSe, version 1.1.01) [33], with an LDA threshold of 2.0. The LEfSe results were validated using the Analysis of Composition of Microbiomes (ANCOM) [34]. Predictive accuracy of discriminative features was evaluated through receiver operating characteristic (ROC) curve analysis, and combined ROC models of bacterial features were generated using CombiRO [35]. Relationships were evaluated using Spearman ρ correlation test. Pair-wise differences were assessed by Mann-Whitney *U* test. Two-way ANOVA with mixed-effects modeling was used to test the impact of time point and group interaction effects. GraphPad Prism (version 9.0) software was used.

Functional prediction of microbial communities was performed using the Phylogenetic Investigation of Communities by Reconstruction of Unobserved States Version 2 (PICRUSt2) version 2.5.0 [36]. Predicted pathway abundance values were reformatted for downstream analysis in LEfSe, which was used to identify significant pathways between groups and across time points using the Kruskal-Wallis and Wilcoxon tests with LDA threshold of 2.0. Functional-taxonomic linkage was assessed using integrative correlation analysis. Spearman rank correlation was applied between enriched microbial taxa and predicted functional pathways from PICRUSt2. Statistical significance was set as *P* < .05.

### Validation on Healthy Donor Oral Microbiome

Microbiome data from an independent age- and sex-matched healthy controls (n = 13) were used for validation. All saliva samples were collected and processed using the same protocols [37] as for the patients with CHB, including DNA extraction prior to whole-genome sequencing. For cross-cohort comparisons, the datasets were harmonized through normalization and alignment; shared species served as the anchor for normalization. Read counts were scaled to comparable levels across datasets to preserve relative abundance. Species unique to each dataset were grouped into an “others” category, thereby maintaining total read depth while avoiding bias from dataset-specific taxa. This approach ensured that all species were represented at a normalized level suitable for cross-cohort analysis. Key species identified in both cohorts were subjected to hierarchical clustering and heatmap visualization for evaluation of relationships across the groups.

### Random Forest Models for Early Outcome Prediction

A predictive model of the final year outcome (favorable vs unfavorable) was developed using clinical or microbiome data from the first 6 months of follow-up. Prespecified feature sets evaluated were: (1) clinical variable only (baseline markers: ALT, aspartate transaminase [AST], bilirubin, HBsAg, HBV DNA); (2) microbiome only (ANCOM-validated microbial markers); and (3) combined clinical plus microbiome markers. Models were trained with random forest classifiers (700 trees, class weighting) and assessed by stratified cross-validation (2–5 folds). Performance was summarized by ROC area under the curve (AUC; primary), balanced accuracy, overall accuracy, and macro F1 score, with confusion matrices for interpretability. Permutation importance was computed for the combined model. As sensitivity analyses, we fit penalized logistic regression (ridge for clinical, elastic-net for microbiome and joint models) to estimate odds ratios with bootstrap confidence intervals. All analyses were conducted in Python 3.9 (scikit-learn) within a Jupyter Notebook environment.

## RESULTS

### Cohort Description and Outcomes at End of Follow-up

Baseline characteristics of the patients with CHB included in this follow-up study of NUC discontinuation are summarized in Table 1. Patient outcomes at the end of follow-up (36 months) were classified as either favorable (n = 8) or unfavorable (n = 10). Independently, participants were categorized as experiencing a hepatic flare or not (Table 2).

**Table 1.** Baseline Characteristics of Study Participants Based on Their Clinical Outcome After Cessation of Nucleos(t)ide Analogue Treatment

Characteristic	Total (n = 18)	Favorable (n = 8)	Unfavorable (n = 10)	*P* Value
**Patient demographics**				
Age, y, mean (SD)	48 (11)	51 (12)	46 (10)	.30
Sex, male, %	72.2	87.5	60	.31
BMI, median (IQR)	26.2 (25.0–29.7)	26.3 (24.3–30.5	26.2 (25.15–28.1)	.37
Ethnicity				
Asian, n (%)	5 (27.8)	1 (12.5)	4 (40)	.31
non-Asian, n (%)	13 (72.2)	7 (87.5)	6 (60)	
ALT, IU/L, mean (SD)	34.8 (12)	34.8 (12)	33.6 (18)	.62
AST, IU/L, mean (SD)	28.1 (6)	27.8 (8)	28.4 (6)	.86
Alcohol usage, glass/wk, mean (SD)	1.1 (2.6)	0.9 (1.1)^a^	1 (2.3)	.47
Tobacco usage, ratio yes:no	0.3:1	0.17:1^a^	0.43:1	.39
Antibiotic usage, ratio yes:no	0.1:1	0:1^a^	0.1:1	.18
**Virological parameters**				
Previous NUC duration, mo, mean (SD)	77 (37)	92 (35)	61 (37)	.15
Previous NUC type				
Entecavir, n (%)	9 (50)	5 (62.5)	4 (40)	.64
Tenofovir, n (%)	9 (50)	3 (37.5)	6 (60)	
HBV DNA undetectable, %**^b^**	100	100	100	1
HBsAg, IU, median (IQR)	1365 (3333)	**558** (**1239)**	**2548** (**4488)**	.**009**
HBsAg, log, median (IQR)	3.14 (0.8)	2.75 (2.2)	3.20 (0.7)	.17

Mann-Whitney *U* test and *t* test ware applied to compare continuous variables, Pearsons χ^2^ and Fisher exact test for categorical variables. Bolded values indicate statistically significant difference.

Abbreviations: ALT, alanine transaminase; AST, aspartate transaminase; BMI, body mass index; HBsAg, hepatitis B surface antigen; HBV, hepatitis B virus; IQR, interquartile range; NUC, nucleos(t)ide analogue.

^a^Data from 1 patient missing.

^b^HBV DNA < 10 IU/mL.

**Table 2.** Baseline Characteristics of Participants With and Without Hepatic Flare

Characteristic	No Flare (n = 8)	Flare (n = 10)	*P* Value
**Patient demographics**			
Age, y, mean (SD)	44 (10)	51 (11)	.20
Sex, male, %	62.5	80	.61
BMI, median (IQR)	26.2 (1.6)	26.7 (7.3)	.54
Ethnicity			
Asian, n (%)	4 (50)	1 (10)	.12
non-Asian, n (%)	4 (50)	9 (90)	
ALT, IU/L, mean (SD)	30.6 (30)	37.2 (12)	.41
AST, IU/L, mean (SD)	25.9 (7)	29.9 (6)	.21
Alcohol usage, glass/wk, mean (SD)	1.4 (2.4)	0.5 (0.9)^a^	.90
Tobacco usage, ratio yes:no	0.33:1	0.29:1^a^	.65
Antibiotic usage, ratio yes:no	0:1	0.14:1^a^	.24
**Virological parameters**			
Previous NUC duration, mo, mean (SD)	70 (46)	80 (31)	.47
Previous NUC type			
Entecavir, n (%)	4 (50)	5 (50)	1
Tenofovir, n (%)	4 (50)	5 (50)	
HBV DNA undetectable, %^b^	100	100	1
HBsAg, IU/mL, median (IQR)	1601 (3716)	1203 (3333)	.90
HBsAg, log IU/mL, median (IQR)	3.20 (1.0)	3.08 (0.9)	.90

Hepatic flare was defined as ALT increase > 80 IU/mL or > 2 × baseline after cessation of nucleos(t)ide analogue treatment. Mann-Whitney *U* test and *t* test ware applied to compare continuous variables, Pearsons χ^2^ and Fisher exact test for categorical variables.

Abbreviations: ALT, alanine transaminase; AST, aspartate transaminase; BMI, body mass index; HBsAg, hepatitis B surface antigen; HBV, hepatitis B virus; IQR, interquartile range; NUC, nucleos(t)ide analogue.

^a^Data from 1 patient missing.

^b^HBV DNA level < 10 IU/mL.

Among baseline variables (demographic, biochemical, virological, and oral health related), the HBsAg level prior to stopping NUC therapy (baseline) was the only parameter significantly associated with an unfavorable outcome (*P* = .009). Hepatic flare (ALT > 2 × upper limit of normal or > 2 × baseline) during follow-up was linked to more frequent meals and higher sweet intake (Supplementary Table 1). At the end of follow-up, all patients with favorable outcomes had an off-therapy viral load < 2000 IU/mL; 50% showed a significant decline or loss of HBsAg, 37.5% achieved seroconversion, and none required NUC reinitiation (Table 3).

**Table 3.** Virological and Biochemical Parameters of Study Participants With Favorable or Unfavorable Outcomes 36 Months After Cessation of Nucleos(t)ide Analogue Treatment

Parameters	Favorable (n = 8)	Unfavorable (n = 10)	*P* Value	No Flare (n = 8)	Flare (n = 10)	*P* Value
HBV DNA, IU/mL, median (SD)	413 (672)	9609 (13 700)	.27	3621 (10 827)	7653 (1113)	.**04**
HBsAg loss, % of participants	**50**	**0**	.**02**	12.5	30	.59
HBsAg, IU/mL, median (IQR)	**105 (481)**	**1647 (4169)**	.**006**	996 (2971)	559 (3045)	.84
Anti-HBs positive, % of participants	37.5	0	.07	20	12.5	1
Restart of NUC, ratio yes:no	**0:8**	**7:3**	.**004**	2:6	5:5	.37
ALT, IU/L, mean (SD)	43.9 (28.3)	31.3 (8.4)	.98	29.5 (9.04)	42.8 (25.3)	.28
AST, IU/L, mean (SD)	40.4 (20.5)	27.1 (8.4)	.53	25.3 (3.6)	38.5 (19.8)	.43

Mann-Whitney *U* test was used for continuous variables, and Fisher exact test for categorical variables. One patient (in the unfavorable outcome group) was followed for 21 mo. Bolded values indicate statistically significant difference.

Abbreviations: ALT, alanine aminotransferase; AST, aspartate aminotransferase; HBsAg, hepatitis B surface antigen; HBV, hepatitis B virus; IQR, interquartile range; NUC, nucleos(t)ide analogue.

### Microbiome Factors Associated With Favorable Outcome or Hepatic Flare

**Figure 1.**
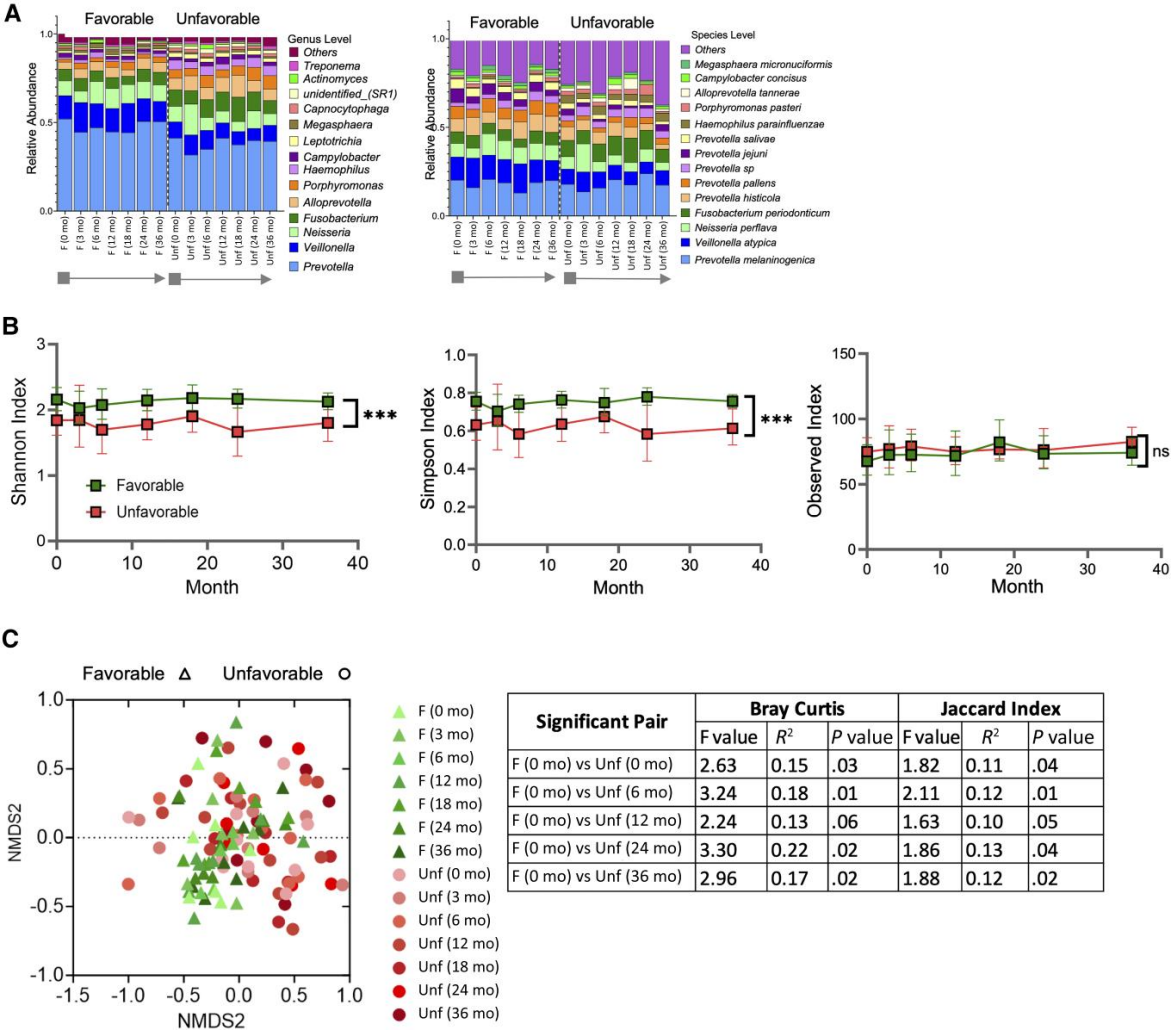
Microbial composition and diversity over time in patients with favorable or unfavorable outcomes. *A*, Relative bacterial abundance at the genus (left) and species (right) levels over time, stratified by clinical outcome, shown as stacked bar plots. Grey box indicates the end of NUC treatment (baseline sample), and the arrow marks the period without NUC treatment and subsequent sampling time points. *B*, Alpha-diversity (Shannon, Simpson, and observed indices) comparisons at species level showing significant differences between favorable and unfavorable outcomes, from baseline to the end of follow-up. Each square represents the group geometric mean at a given time point, and bars are 95% confidence intervals. Green indicates the favorable group; red indicates the unfavorable group. *C*, Beta-diversity assessed by Bray-Curtis and Jaccard indices visualized in NMDS plot, in which individual samples are labeled with clinical outcome and time point. ***P < .001 by 2-way ANOVA with mixed-effects modeling. Abbreviations: F, favorable; NMDS, nonmetric multidimensional scaling; NUC, nucleos(t)ide analogue; ns, not significant; Unf, unfavorable.

The oral microbiome of each patient was characterized by sequencing saliva samples (n = 110) collected from baseline to the end of follow-up across 7 time points. As shown in Figure 1*A* and 1*B*, overall microbiome diversity and taxonomic composition remained stable over time within each outcome group. Interestingly, patients with a favorable outcome consistently exhibited greater richness and diversity than those with an unfavorable outcome (Shannon and Simpson indices, *P* < .001). Between sample variation (Bray-Curtis and Jaccard beta diversity) was also significant (*P* = .001; Figure 1*C*, left) and when comparing the favorable at 0 months with unfavorable at different time points (Figure 1*C*, right).

**Figure 2.**
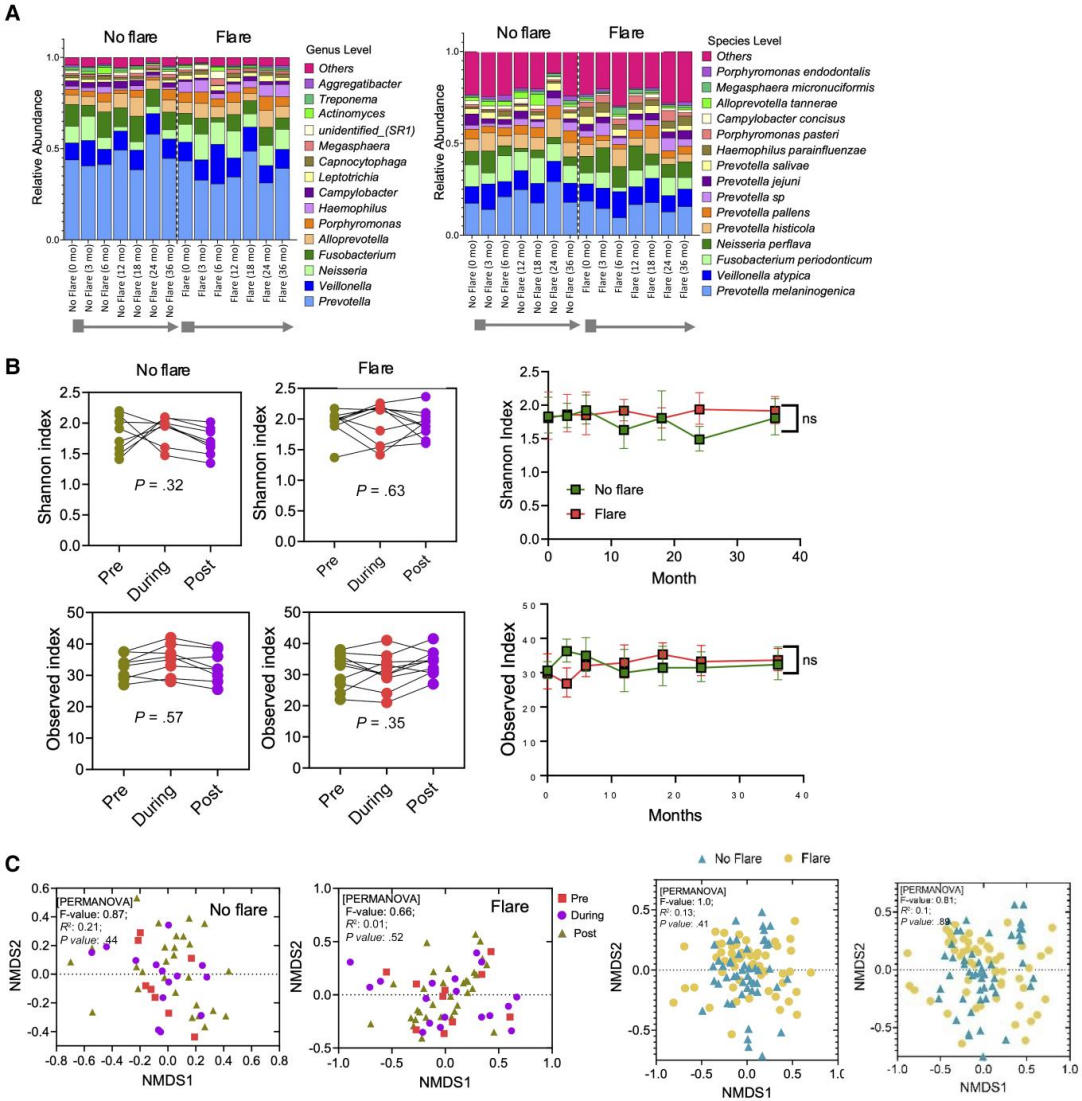
Microbial composition and diversity over time in patients with or without flare during follow-up. *A*, Stacked bar plots of relative bacterial abundance at the genus (left) and species (right) levels over time in patients without flare and with flare. *B*, Alpha diversity indices with paired comparisons at pre-, during-, and postflare phases (or equivalent time points in patients without flare) Error bars indicate 95% CI. *C*, Beta diversity assessed at the species level, shown as NMDS ordinations, comparing community composition between flare and no-flare groups across pre-, during-, and postflare phases, as well as overall comparisons using all samples combined within each group (flare vs no flare). Abbreviations: NMDS, nonmetric multidimensional scaling; ns, not significant.

We next investigated whether oral microbiome shifts distinguished participants who experienced an ALT flare, as classified in Table 2. Eight patients developed a flare, 6 of which occurred within the first 200 days, specifically on days 64, 80, 84, 170, 187, and 192, while 2 additional were recorded on days 317 and 561 (Figure 2*A*). To study potential flare-related microbiome changes, samples from patients with no flare were time matched to the flare cohort (preflare, flare, and postflare). This alignment allowed direct comparison of microbiome dynamics during flares but revealed no consistent interpatient change in oral microbiome diversity (Figure 2*B*, left). Excluding the flare samples in the analysis revealed no significant difference in alpha diversity between the groups (Figure 2*B*, right). Multidimensional scaling plots also showed no significant separations between flare and no-flare samples (Figure 2*C*).

### Distinct Microbiome Markers Correlate With Virological and Biochemical Variables

**Figure 3.**
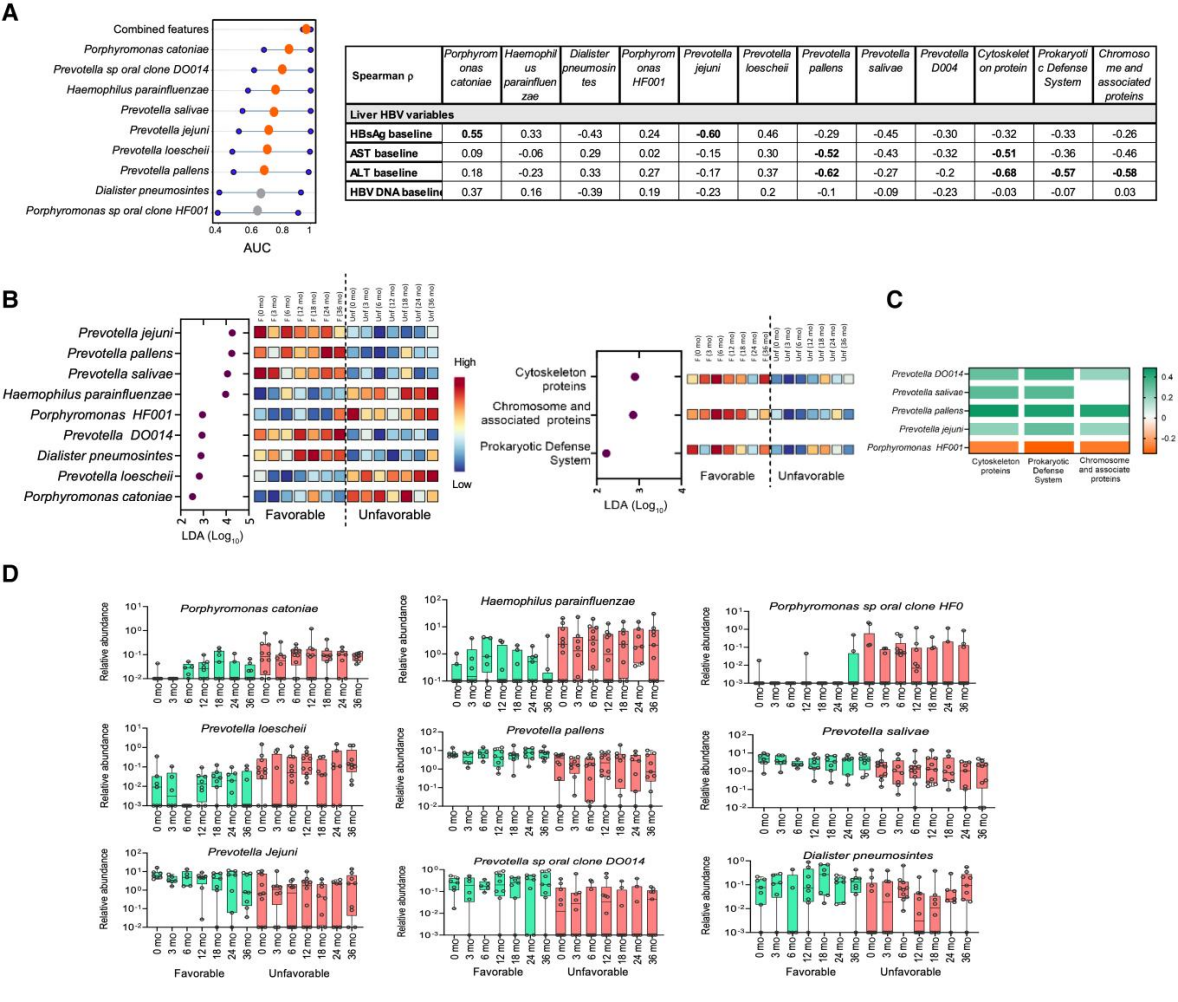
Distinctive microbial signatures associated with favorable and unfavorable outcomes. *A*, Left, biomarker analysis at the species level of the baseline microbiome. Individual and combined marker taxa are shown with ROC AUC scores. Right, Spearman correlation analysis of baseline samples identified specific taxa correlating with HBV or liver enzyme markers (bold, ρ > 0.5, *P* < .05). *B*, Left, LEfSe analysis identified significant microbial species signatures (LDA > 0.2, *P* < .05). Right, putative functional pathways (PICRUSt2; LDA score > 2.0, *P* < .05) across 7 time points associated with either favorable or unfavorable outcomes. *C*, Significant microbial taxa output linked to functional pathways (*P* < .05). *D*, Relative abundances of specific taxa over time in favorable (green) and unfavorable (red) groups. Box indicates the 25th to 75th percentiles. The line in the middle of the box indicates the median. Whiskers represent minimum to maximum values. X-axis, sample time points; Y-axis, relative abundance of respective microbial feature. Abbreviations: HBV, hepatitis B virus; LDA, linear discriminant analysis; LEfSe, linear discriminant analysis effect size; PICRUSt2, Phylogenetic Investigation of Communities by Reconstruction of Unobserved States, version 2; ROC AUC, receiver operating characteristic area under the curve.

Given that the oral microbiome profile appeared stable but outcome specific, we next evaluated its biomarker potential. In baseline samples (Figure 3*A*), a combined panel comprising *Haemophilus parainfluenzae* and *Porphyromonas catoniae* together with several *Prevotella* species such as *Prevotella pallens, Prevotella* sp oral clone DO014*, Prevotella jejuni, Prevotella salivae, Prevotella loescheii, Porphyromonas* sp oral clone HF001, and *Dialister pneumosintes* yielded ROC AUC = 0.98 (*P* = .0001). At the same time point, *P. catoniae* abundance correlated positively with HBsAg levels (ρ = 0.55, *P* < .01), whereas *P. jejuni* and *P. pallens* correlated negatively with HBsAg and with AST and ALT, respectively.

Linear discriminant analysis of the time points after NUC cessation (Figure 3*B*, left) showed consistent enrichment of several *Prevotella* spp (*P. pallens, Prevotella* sp oral clone DO014, *P. jejuni, P. salivae*) in the favorable group (LDA > 2, *P* < .05), while the unfavorable group was enriched in *H. parainfluenzae*, *Prevotella* oral clone, *P. loescheii*, and *P. catoniae* (LDA > 2; *P* < .05). In subsequent ROC validation analysis, the combined baseline microbial features yielded strong discriminatory performance (0.87–1.00, *P* ≤ .001) across all subsequent sample time points (Supplementary Table 2). Moreover, the favorable group displayed persistent enrichment of pathways related to prokaryotic defense systems, chromosomal associated proteins, and cytoskeletal proteins, with steady abundances of these identified microbial features across the study period (Figure 3*B*, right). By extracting taxa-level functional contributions from the PICRUSt2 predictions, we found that these functional pathways were primarily driven by *P. jejuni, P. pallens, P. salivae,* and *Prevotella* DO014 (ρ = 0.20–0.49, *P* < .05), while *Porphyromonas* HF001 showed an inverse association (ρ = −0.27 to −0.35, *P* < .05) (Figure 3*C*).

**Figure 4.**
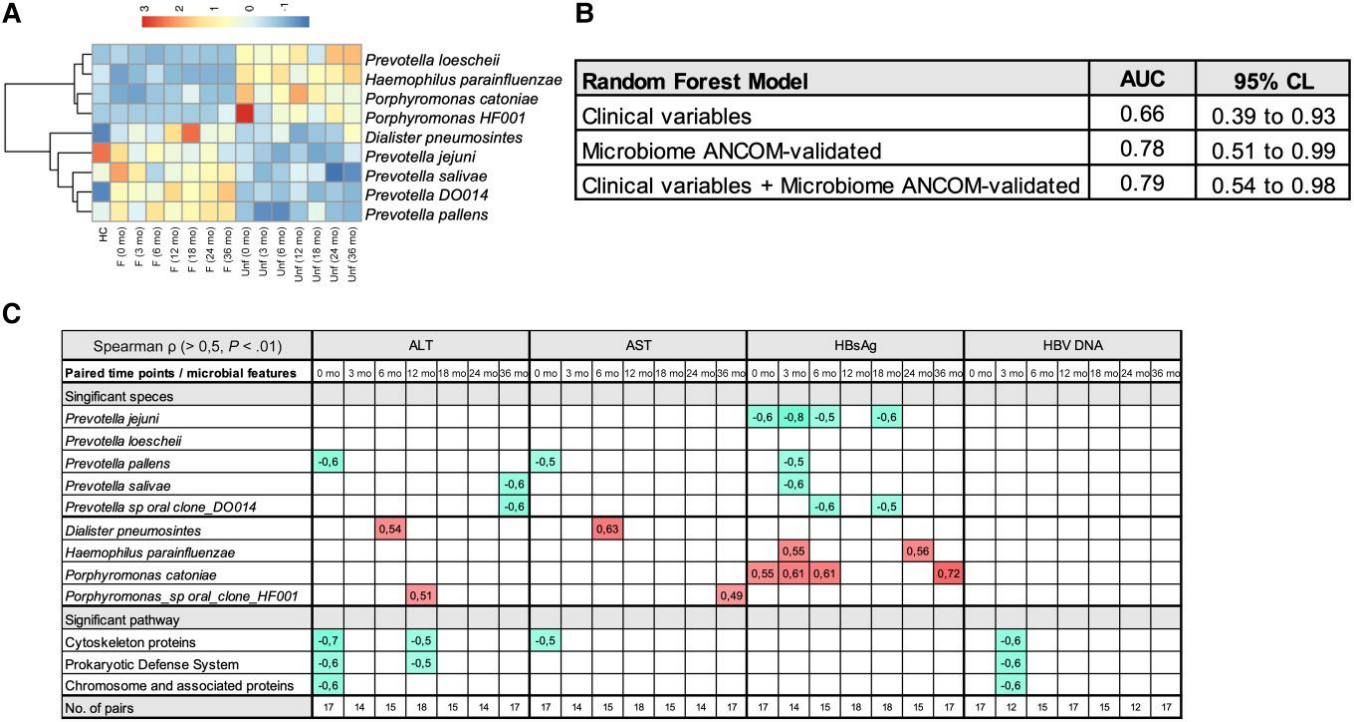
Predictive modeling of clinical outcomes using early ANCOM-validated microbiome and clinical data. *A*, Hierarchical clustering heatmap of identified features, revealing 2 main bacterial clusters: one enriched in the unfavorable group, the other enriched in the favorable group, together with sex- and age-matched healthy controls (n = 13). *B*, Random forest models for early prediction (outcome: favorable or unfavorable) using data from the first 6 months of follow-up. Three feature sets evaluated were: (1) clinical variables only (ALT, AST, bilirubin, HBsAg, HBV DNA), (2) microbiome markers only (ANCOM-validated ≤ 6 mo), and (3) combined clinical + microbiome markers. Models were trained with random forest classifiers (700 trees, class weighting), and performance was assessed using AUC with 95% CI. *C*, Association between microbial features and HBV and liver enzyme markers at each paired time points (Spearman ρ > 0.5, *P* < .01, are shown). Abbreviations: ANCOM, Analysis of Composition of Microbiomes; F, favorable; HC, healthy control; Unf, unfavorable.

To improve the generalizability of the exploratory applicability to broader clinical scenarios, an independent cohort of healthy controls was used to validate the identified microbiome features. As shown in Figure 4*A*, a heatmap of hierarchical clustering revealed 2 main bacterial clusters. One cluster with overrepresentation of *H. parainfluenzae, P. catoniae, P. loescheii,* and *Porphyromonas* sp HF001 infrequently seen in the favorable or healthy control samples, in line with the relative abundance levels in the respective patient groups (Figure 3*D*). The other cluster displayed a steady pattern of *P. pallens, P. jejuni, P. salivae, Prevotella* sp DO014, and *D. pneumosintes*, and was frequently seen in the favorable group and healthy donors.

The clinical applicability of the microbiome marker concept was then challenged to test its utility in identifying early predictors of outcome. For this, we used results of the clinical and microbial variables available from the first 6 months of the study, in which the most significant taxa (ANCOM validated) were used, that is *P. salivae, P. pallens, Prevotella* DO014*, P. catoniae, P. jejuni,* and *H. parainfluenzae.* (Supplementary Table 3). As shown in Figure 4*B*, random forest analysis revealed that microbiome markers improved outcome prediction over clinical variables alone; the clinical variable-only model showed modest performance (AUC = 0.663; balanced accuracy = 0.650; F1 = 0.649), while AUC was improved in the microbiome-only model (AUC = 0.775; balanced accuracy = 0.725; F1 = 0.721), as well as in the combined clinical plus microbiome model (AUC = 0.787; balanced accuracy = 0.713; F1 = 0.714), and thus was capable of predicting 62% of favorable and 80% of unfavorable outcomes.

Longitudinal correlation analysis (Figure 4*C*) further identified that the *Prevotella* species repeatedly correlated negatively with clinical CHB markers (ρ > 0.5, *P* < .01), which support their association with lower viral and biochemical levels. Conversely, strong positive correlations between *P. catoniae*, *H. parainfluenzae,* and HBsAg were noted both at study start and toward the end of follow-up. To evaluate whether potential covariates (eg, oral health and lifestyle factors) may have influenced our findings, a network-based Spearman correlation analysis was conducted, revealing no significant associations with any of the identified microbial markers (Supplementary Table 4).

## DISCUSSION

This long-term longitudinal study is the first to characterize the oral microbiome in patients with CHB who discontinue NUC therapy. Higher oral microbiome diversity prior to cessation of NUC therapy (here our baseline) and throughout the follow-up correlated with a favorable clinical outcome, that is reduced or lost HBsAg, sustained virological suppression in year 3, and no need to restart NUC therapy. This highlights the potential of the oral microbiome as a predictive proxy for NUC treatment cessation success. Our results represent a novel concept of exploring the oral microbiome and its influence on outcomes after NUC cessation, a first step toward integrating microbiome profiling into long-term outcome stratification for CHB. This underscores the importance of investigating the involvement of the microbiome in the mechanisms underlying spontaneous HBV clearance without antiviral therapy.

Although hepatic flares were common after NUC withdrawal, they did not measurably alter the oral microbiome diversity seen in our study participants. One explanation may be the flare definition (ALT > 80 U/L or > 2 × baseline). In other cohorts, higher-grade flares after NUC cessation have been reported [10]; therefore, the microbiome impact in those patients remains to be investigated. The absence of intraindividual microbiome shifts during flare episodes in our study suggests these flares are immunologically, not microbially, driven, supported by the lack of consistent taxonomic or diversity changes despite precise temporal alignment of samples.

Distinct microbial signatures further separated outcome groups. *Prevotella* spp were consistently enriched in patients with favorable outcomes and correlated negatively with HBsAg, ALT, and AST at multiple time points. Conversely, taxa such as *H. parainfluenzae* and *P. catoniae* were enriched in the unfavorable outcome group and correlated positively with HBV-specific markers. Functional profiling showed enrichment of pathways linked to host defense and structural integrity in the favorable outcome group, suggesting a protective microbiome configuration Our result is timely, given the increasing recognition of the consequences of dysbiosis in the development of cirrhosis, where poor oral health can further exacerbate liver disease and immune dysfunction [38]. Early dysfunction of the oral-gut-liver axis therefore merits increased clinical attention, and the noninvasive nature of saliva sampling makes it applicable for prognostication in outpatient care beyond CHB [22].

Our observation that a resilient, *Prevotella*-enriched oral microbiome accompanies successful NUC discontinuation aligns with emerging evidence. *Prevotella* species are generally regarded as commensals in the oral cavity. In this regard Lee et al [18 ] recently reported that not only the richness of the microbiome increased from decompensated to stable cirrhosis to the highest in healthy individuals, but also *Prevotella* spp are enriched in SAL1 (a dominant saliva type in healthy controls without cirrhosis), and key salivary commensals that reduce likelihood of hospitalization in patients with cirrhosis [22]. Moreover, in a study of the gut microbiome impact on virological responses to NUC treatment, the *Prevotella* cluster appeared as one of the significant major clusters [14]. Another recent observation in HBeAg-negative individuals who had achieved functional cure indicates that gut microbes producing short-chain fatty acids contribute to butyrate-mediated inhibition of HBV production in vitro [39]. Consistent with these studies, our results underscore the importance of the digestive microbiome, in which both oral and gut health could contribute to clearance of HBV, and is in line with the accumulating evidence that the immunity competence relies on host microbiome health [21].

Although the targeted 16S rRNA approach is known to limit resolution to the genus level, classification rates are highly dependent on the reference database and environment. Oral microbiome coverage has markedly improved in recent years: Caselli et al [40] in 2020 reported > 700 oral species with approximately 54% cultivated, and the extended Human Oral Microbiome Database (HOMD) (version 4.1) now catalogs 836 taxa, of which approximately 70% have been named or cultivated. Recent advances in genome-based taxonomies and full-length 16S reference sets also have further expanded species-level resolution for oral taxa. More recent studies have also consistently applied QIIME for species-level resolution in various disease cohorts [41–46]. The rapid development and turnover in reference databases thus make targeted oral microbiome analysis relevant for clinical microbiome applications.

While this study provides new insights, several limitations should be noted. First, this small cohort is underpowered, limiting the generalizability of our findings; the generated models should be regarded as exploratory rather than definitive predictors. Future validation using larger and independent cohorts, with cross-validations and more refined methods such as long-read sequencing, will be required to confirm the predictive accuracy of these microbial features. Second, our study focuses solely on the oral microbiome to promote patient compliance; additional oral-gut microbiome comparisons could provide more insights. Finally, as our study is observational, causative links between microbiome shifts and treatment outcomes remain to be confirmed with new studies.

Our findings suggest potential pathways by which the oral microbiome may influence outcome in planned HBV NUC cessation. Further investigation is required to determine whether microbiome modulation could influence CHB progression and functional cure, or reduce undefined inflammatory drivers as observed in cirrhosis [22]. Improved prediction performance with oral microbiome data suggests early microbial profiling offers added prognostic information.

## Supplementary Material

jiaf591_Supplementary_Data

## Supplementary Data

Supplementary materials are available at *The Journal of Infectious Diseases* online (http://jid.oxfordjournals.org/). Supplementary materials consist of data provided by the author that are published to benefit the reader. The posted materials are not copyedited. The contents of all supplementary data are the sole responsibility of the authors. Questions or messages regarding errors should be addressed to the author.
