# Supplementary material for: Oral Microbiome Diversity Matters on Nucleos(t)ide Analogue Cessation in Chronic Hepatitis B
Source: J Infect Dis. 2025 Dec 2;233(3):e630–40. doi: 10.1093/infdis/jiaf591 (PMC13017730; doi:10.1093/infdis/jiaf591)
Supplement: jiaf591_Supplementary_Data [file jiaf591_supplementary_data.zip › Supplemantary Table 1-2.docx]

| **Supplementary Table 1. Oral health evaluation of study participants at baseline.** | | | | | | |
| --- | --- | --- | --- | --- | --- | --- |
| **Oral health factors** | **Favorable** ^a^ **(n=8)** | **Un/ favorable (n=10)** | **P-value** | **No flare**  **(n=8)** | **Flare** ^a^ **(n=10)** | **P-value** |
| Teeth brushing (≥2/day), (%) | 43 | 70 | 0.33 | 63 | 56 | 0.48 |
| Gum bleeding (≥1/week), (%) | 71 | 60 | 0.46 | 75 | 56 | 0.46 |
| Have gum disease, (%) | 17 | 20 | 0.69 | 13 | 29 | 0.32 |
| Treatment for gum disease, (%) | 33 | 30 | 0.37 | 14 | 33 | 0.42 |
| Have bone loss, (%) | 14 | 0 | 0.33 | 14 | 0 | 0.33 |
| Have loose teeth, (%) | 14 | 10 | 0.50 | 13 | 11 | 0.65 |
| Teeth does not look right, (%) | 0 | 10 | 0.33 | 0 | 11 | 0.41 |
| Experienced oral health (≥good), (%) | 71 | 40 | 0.36 | 63 | 44 | 0.56 |
| Vegeterian, (%) | 0 | 0 | 0.44 | 0 | 0 | 1 |
| Meals (#daily freq.), (median, IQR) | 3 (3) | 3 (2) | 0.76 | **2 (1)** | **4 (2)** | **0.04** |
| Sweet intake (#weekly freq.), (median, IQR) | 2 (5) | 2 (3) | 0.88 | **1 (1)** | **3 (10)** | **0.02** |
| Use dental floss, (%) | 33 | 22 | 0.51 | 17 | 33 | 0.67 |
| Use mouthwash, (%) | 0 | 30 | 0.09 | 43 | 0 | 0.09 |
| *Mann–Whitney U test was applied to compare the continuous variables, Pearsons Chi-Square and Fisher's Exact Test for categorical variables between Favourable vs Unfavourable and Non-flare vs Flare patient groups.*  *^a^ data from one patient missing* | | | | | | |

**Supplementary Table 2.
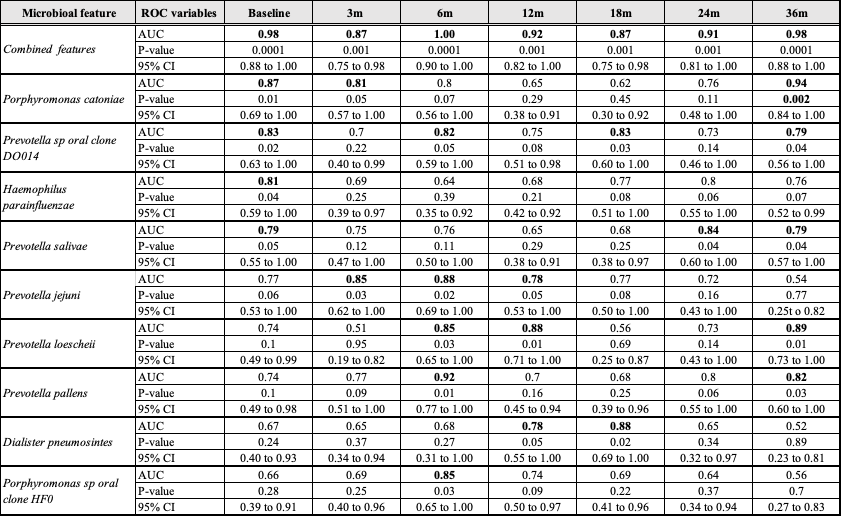
** **Baseline combined or single microbial features and the yielded area under curve (AUC) discriminatory performance scores on each sample timepoint.**
