# Supplementary material for: Oral Microbiome Diversity Matters on Nucleos(t)ide Analogue Cessation in Chronic Hepatitis B
Source: J Infect Dis. 2025 Dec 2;233(3):e630–40. doi: 10.1093/infdis/jiaf591 (PMC13017730; doi:10.1093/infdis/jiaf591)
Supplement: jiaf591_Supplementary_Data [file jiaf591_supplementary_data.zip › Supplementary Table 3.docx]

**Supplementary Table 3. ANCOM analysis of species-level differential abundance**

| **Species** | **Log Fold Change (lfc)** | **Standard Error (se)** | **Test Statistic (W)** | **Raw p-value (p)** | **Adjusted q-value (q)** | **Differentially Abundant (diff)** | **Passed Structural Zeros (passed_ss)** | **Differentially Abundant (Robust) (diff_robust)** |
| --- | --- | --- | --- | --- | --- | --- | --- | --- |
| *Prevotella salivae* | -1,26 | 0,27 | -4,61 | 1.15e-05 | 9.85e-05 | TRUE | TRUE | TRUE |
| *Prevotella pallens* | -1,61 | 0,3 | -5,39 | 4.63e-07 | 8.38e-06 | TRUE | TRUE | TRUE |
| *Prevotella DO014* | -1,12 | 0,22 | -5,21 | 1.80e-06 | 2.44e-05 | TRUE | TRUE | TRUE |
| *Porphyromonas catoniae* | 0,42 | 0,16 | 2,57 | 0.0124 | 0.0350 | TRUE | TRUE | TRUE |
| *Prevotella jejuni* | -0,89 | 0,3 | -2,99 | 0.00359 | 0.0127 | TRUE | TRUE | TRUE |
| *Haemophilus parainfluenzae* | 0,74 | 0,21 | 3,51 | 0.000808 | 0.00387 | TRUE | TRUE | TRUE |
| *Dialister pneumosintes* | -0,77 | 0,21 | -3,65 | 0.000456 | 0.00240 | TRUE | FALSE | FALSE |
| *Porphyromonas HF001* | -0,04 | 0,18 | -0,24 | 0.813 | 0.902 | FALSE | FALSE | FALSE |
| *Prevotella loescheii* | 0,91 | 0,2 | 4,46 | 2.87e-05 | 0.000213 | TRUE | FALSE | FALSE |
