# Supplementary material for: Oral Microbiome Diversity Matters on Nucleos(t)ide Analogue Cessation in Chronic Hepatitis B
Source: J Infect Dis. 2025 Dec 2;233(3):e630–40. doi: 10.1093/infdis/jiaf591 (PMC13017730; doi:10.1093/infdis/jiaf591)
Supplement: jiaf591_Supplementary_Data [file jiaf591_supplementary_data.zip › Supplementary Table 4.docx]

| **Baseline bacterial**  **features** | **Spearman’s rank**  **correlation** | **BMI** | **Sex** | **Age** | **Antibiotics** | **Tobaco** | **Glas of alcohol** | **Brushing**  **frequency** | **Bleeding gum** | **Sweet intake** | **Gum disease** | **Self-rated oral**  **health** | **Periodontal treatment** | **Gingivitis treatment** | **Loose teeth** | **Bone loss** |
| --- | --- | --- | --- | --- | --- | --- | --- | --- | --- | --- | --- | --- | --- | --- | --- | --- |
| *p.catoniae* | rho | 0.12 | 0.24 | -0.24 | 0.09 | 0.11 | 0.2 | -0.08 | -0.02 | 0.61 | -0.03 | 0.04 | -0.18 | -0.18 | -0.1 | -0.24 |
|  | 95% CI | -0.41 to 0.59 | -0.29 to 0.65 | -0.66 to 0.28 | -0.44 to 0.57 | -0.41 to 0.57 | -0.33 to 0.63 | -0.55 to 0.43 | -0.51 to 0.47 | 0.15 to 0.85 | -0.51 to 0.47 | -0.46 to 0.52 | -0.62 to 0.34 | -0.62 to 0.34 | -0.56 to 0.41 | -0.66 to 0.28 |
|  | P-value | 0.65 | 0.38 | 0.35 | 0.94 | 0.68 | 0.43 | 0.75 | 0.93 | 0.92 | 0.88 | 0.5 | 0.5 | 0.75 | 0.71 | 0.66 |
| *H.parainfluenzae* | rho | 0.44 | 0.41 | 0.31 | 0.38 | 0.1 | 0.14 | 0.17 | 0.35 | 0.43 | 0.02 | 0.11 | 0.07 | 0.07 | 0.28 | 0 |
|  | 95% CI | -0.09 to 0.77 | -0.1 to 0.75 | -0.21 to 0.7 | -0.16 to 0.74 | -0.41 to 0.57 | -0.38 to 0.59 | -0.36 to 0.61 | -0.17 to 0.72 | -0.09 to 0.77 | -0.47 to 0.51 | -0.4 to 0.57 | -0.44 to 0.54 | -0.44 to 0.54 | -0.25 to 0.68 | -0.49 to 0.49 |
|  | P-value | 0.09 | 0.11 | 0.22 | 0.13 | 0.7 | 0.62 | 0.53 | 0.17 | 0.94 | 0.66 | 0.8 | 0.8 | 0.38 | 1 | 0.38 |
| *D.pneumosintes* | rho | 0 | -0.1 | 0.06 | -0.26 | -0.19 | 0.04 | -0.47 | -0.36 | -0.18 | 0.07 | 0.07 | 0.28 | 0.18 | -0.19 | 0 |
|  | 95% CI | -0.51 to 0.51 | -0.57 to 0.41 | -0.45 to 0.54 | -0.68 to 0.28 | -0.62 to 0.33 | -0.46 to 0.52 | -0.78 to 0.03 | -0.72 to 0.16 | -0.63 to 0.36 | -0.44 to 0.54 | -0.44 to 0.54 | -0.24 to 0.68 | -0.34 to 0.62 | -0.63 to 0.33 | -0.49 to 0.49 |
|  | P-value | 0.99 | 0.72 | 0.82 | 0.69 | 0.47 | 0.94 | 0.06 | 0.16 | 0.8 | 0.81 | 0.27 | 0.48 | 0.5 | 1 | 0.5 |
| *P.HF001* | rho | 0.18 | 0.62 | -0.15 | -0.17 | -0.12 | -0.23 | 0.02 | 0.31 | 0.07 | 0.08 | 0.03 | 0.11 | 0.11 | 0.07 | 0.25 |
|  | 95% CI | -0.36 to 0.63 | 0.19 to 0.85 | -0.6 to 0.37 | -0.62 to 0.37 | -0.58 to 0.4 | -0.65 to 0.29 | -0.48 to 0.51 | -0.22 to 0.7 | -0.46 to 0.55 | -0.43 to 0.55 | -0.47 to 0.51 | -0.4 to 0.57 | -0.4 to 0.57 | -0.44 to 0.54 | -0.27 to 0.66 |
|  | P-value | 0.52 | 0.01 | 0.56 | 1 | 0.62 | 0.65 | 0.96 | 0.22 | 0.87 | 0.93 | 0.77 | 0.77 | 1 | 0.29 | 0.96 |
| *P. jejuni* | rho | -0.37 | -0.43 | 0.04 | -0.37 | 0.13 | 0.07 | -0.15 | -0.13 | -0.64 | -0.02 | -0.16 | 0.11 | 0.18 | -0.19 | 0.1 |
|  | 95% CI | -0.74 to 0.17 | -0.76 to 0.08 | -0.46 to 0.52 | -0.74 to 0.17 | -0.39 to 0.58 | -0.43 to 0.55 | -0.6 to 0.37 | -0.59 to 0.38 | -0.87 to -0.2 | -0.5 to 0.48 | -0.61 to 0.36 | -0.4 to 0.57 | -0.34 to 0.62 | -0.62 to 0.34 | -0.41 to 0.57 |
|  | P-value | 0.16 | 0.1 | 0.89 | 0.31 | 0.63 | 0.82 | 0.57 | 0.6 | 0.96 | 0.53 | 0.67 | 0.48 | 0.53 | 0.82 | 0.55 |
| *P.loescheii* | rho | 0.1 | 0.2 | -0.34 | 0.31 | -0.2 | 0.04 | -0.1 | -0.13 | 0.64 | 0.23 | 0.14 | -0.1 | -0.1 | 0.08 | -0.16 |
|  | 95% CI | -0.43 to 0.58 | -0.32 to 0.63 | -0.71 to 0.19 | -0.23 to 0.71 | -0.63 to 0.33 | -0.46 to 0.52 | -0.56 to 0.41 | -0.59 to 0.39 | 0.2 to 0.87 | -0.29 to 0.65 | -0.38 to 0.59 | -0.56 to 0.42 | -0.56 to 0.42 | -0.43 to 0.55 | -0.6 to 0.37 |
|  | P-value | 0.72 | 0.46 | 0.18 | 0.5 | 0.45 | 0.94 | 0.71 | 0.61 | 0.38 | 0.6 | 0.72 | 0.72 | 0.82 | 0.71 | 0.82 |
| *P.pallens* | rho | -0.23 | -0.11 | 0.06 | 0.03 | 0 | 0.15 | -0.15 | -0.44 | -0.22 | 0.08 | -0.38 | 0.23 | 0.15 | 0.3 | 0.41 |
|  | 95% CI | -0.66 to 0.31 | -0.57 to 0.4 | -0.44 to 0.54 | -0.49 to 0.53 | -0.49 to 0.5 | -0.37 to 0.6 | -0.6 to 0.37 | -0.77 to 0.06 | -0.65 to 0.33 | -0.43 to 0.55 | -0.73 to 0.14 | -0.3 to 0.65 | -0.37 to 0.6 | -0.23 to 0.69 | -0.11 to 0.75 |
|  | P-value | 0.39 | 0.68 | 0.81 | 1 | 0.99 | 0.61 | 0.57 | 0.08 | 0.78 | 0.14 | 0.38 | 0.56 | 0.29 | 0.06 | 0.24 |
| *P.salivae* | rho | -0.19 | -0.48 | 0.2 | -0.08 | 0.33 | -0.19 | -0.25 | -0.22 | -0.19 | 0.08 | -0.25 | 0.2 | 0.25 | 0 | 0.1 |
|  | 95% CI | -0.64 to 0.35 | -0.79 to 0.02 | -0.32 to 0.63 | -0.57 to 0.44 | -0.2 to 0.7 | -0.62 to 0.34 | -0.66 to 0.28 | -0.64 to 0.31 | -0.64 to 0.35 | -0.43 to 0.55 | -0.66 to 0.28 | -0.32 to 0.63 | -0.28 to 0.66 | -0.49 to 0.49 | -0.41 to 0.57 |
|  | P-value | 0.48 | 0.06 | 0.43 | 0.88 | 0.2 | 0.53 | 0.33 | 0.4 | 0.76 | 0.33 | 0.44 | 0.33 | 1 | 0.82 | 0.99 |
| *P.DO014* | rho | -0.39 | -0.41 | 0.05 | -0.31 | 0.29 | -0.04 | -0.15 | -0.32 | -0.49 | 0.03 | -0.24 | 0.01 | 0.11 | -0.23 | 0 |
|  | 95% CI | -0.75 to 0.15 | -0.75 to 0.11 | -0.46 to 0.53 | -0.71 to 0.23 | -0.24 to 0.68 | -0.52 to 0.46 | -0.6 to 0.37 | -0.7 to 0.2 | -0.8 to 0.03 | -0.47 to 0.52 | -0.66 to 0.29 | -0.48 to 0.5 | -0.4 to 0.57 | -0.65 to 0.3 | -0.49 to 0.49 |
|  | P-value | 0.14 | 0.12 | 0.86 | 0.5 | 0.27 | 0.94 | 0.57 | 0.21 | 0.92 | 0.35 | 0.96 | 0.67 | 0.44 | 1 | 0.44 |

**Supplementary Table 4. Spearman correlation analysis between microbial markers and oral hygiene/lifestyle covariates**
